# Addressing parachute research and removing barriers for LMIC researchers in *Microbial Genomics*

**DOI:** 10.1099/mgen.0.000722

**Published:** 2021-10-27

**Authors:** Eva Heinz, Kathryn E. Holt, Conor J. Meehan, Samuel K. Sheppard

**Affiliations:** ^1^​ Departments of Vector Biology and Clinical Sciences, Liverpool School of Tropical Medicine, Liverpool, UK; ^2^​ London School of Hygiene & Tropical Medicine, London WC1E 7HT, UK; ^3^​ School of Chemistry and Biosciences, University of Bradford, Bradford, UK; ^4^​ Unit of Mycobacteriology, Institute of Tropical Medicine, Antwerp, Belgium; ^5^​ The Milner Centre for Evolution, Department of Biology & Biochemistry, University of Bath, Claverton Down, Bath BA2 7AY, UK

**Keywords:** parachute research, publishing, society publishing, editorial policy

Parachute research (a.k.a. helicopter research) is a commonly used term describing the practice of conducting primary research within a host country and subsequently publishing findings with inadequate recognition of local researchers, staff and/or the supporting infrastructure [1]. This predominantly takes place in Low- and Middle-Income Countries (LMICs), where research funding is sparse and leads to a drastic power imbalance towards researchers from High-Income Countries (HICs). As a consequence, collaborations can result in extractive research, inadequate recognition regarding authorship and no effort to contribute to knowledge transfer or career development opportunities for local partners. An additional driver of inequality in technology-driven fields such as microbial genomics is that sequencing and informatics infrastructure is often based in HICs. This can mean that while specimen and data collection and microbiology are carried out by LMIC partners (or parachuting researchers), the sequencing, analysis and paper writing is often conducted by the HIC partners with little to no effort to include LMIC partners in the planning or analysis stages. Importantly, in the context of infectious disease research, it also impedes the deployment of contemporary genomics surveillance capability in the countries where they are most needed. Despite the ongoing discussion and documentation of parachute research [2], inequities remain even in very recent literature and understandably cause increasing frustration. Every fifth article on COVID in Africa was published without an author from the continent, highlighting the urgent need for clearer actions [3].

Very recently, some independent efforts have been launched to address persistent parachute research (e.g. at PLoS [4]); and especially society journals have a central role in scientific culture. At *Microbial Genomics* we are firmly against parachute research and we seek to implement additional effective actions to combat it. When assessing the recent publications in *Microbial Genomics*, 24 articles focused on LMIC pathogen epidemiology in 2021 and all included local co-authors with 75% including an LMIC author in first and/or last position. We hope to further improve this comparatively positive situation by taking clear action against parachute and exploitative research. One approach being considered is the development of reflexivity statements, similar to an ethics statement, in which authors respond to questions about their contributions in relation to the local (e.g. LMIC) setting, as recently proposed [5]. A related issue especially relevant in the (microbial) genomics field is the exploitation of indigenous communities through extractive research without community engagement, which we also aim to target. Working with Microbiology Society, we plan to establish a policy for implementing the consensus recommendations on reflexivity statements [5]. We will be reaching out to the *Microbial Genomics* community, particularly LMIC researchers and indigenous researchers, to work with us in developing these new guidelines and the format of the reflexivity statement. In the meantime, we note that submissions to Microbiology Society journals including *Microbial Genomics* are encouraged to include author statements, utilising the CRediT taxonomy [6] of contributor roles; and highlight that both these and the ICMJE authorship guidelines [7] should be applied in an inclusive manner rather than used as an excuse to exclude overseas scientists.

As a complementary step to increase accessibility of science for everyone, consistent with the journal’s ethos of pioneering open science, *Microbial Genomics* has a fee-free publishing option [8] for corresponding authors based in countries listed under Group A and B of the Hinari programme, to further empower local researchers and remove financial barriers to publish their work, as well as providing free access to all science published in *Microbial Genomics*. Together with our existing open data policy [9] and occasional sponsorship for LMIC researchers to attend international conferences such as this one [10], we are taking clear and practical measures to promote equity and access to scientific publishing and would like to re-emphasize that we encourage fee-free submissions from LMIC researchers.

## References

[1] The Lancet Global Health (2018). Closing the door on parachutes and parasites. Lancet Glob Health.

[2] Ghani M, Hurrell R, Verceles AC, McCurdy MT, Papali A (2021). Geographic, Subject, and Authorship Trends among LMIC-based Scientific Publications in High-impact Global Health and General Medicine Journals: A 30-Month Bibliometric Analysis. J Epidemiol Glob Health.

[3] Naidoo AV, Hodkinson P, Lai King L, Wallis LA (2021). African authorship on African papers during the COVID-19 pandemic. BMJ Glob Health.

[4] PLOS (2021). Announcing a new PLOS policy on inclusion in global research. The Official PLOS Blog. https://theplosblog.plos.org/2021/09/announcing-a-new-plos-policy-on-inclusion-in-global-research/.

[5] Morton B, Vercueil A, Masekela R, Heinz E, Reimer L (2021). Consensus statement on measures to promote equitable authorship in the publication of research from international partnerships. Anaesthesia.

[6] Microbiology Society (2021). How to prepare an article for submission: Author statements. https://www.microbiologyresearch.org/prepare-an-article#8.

[7] International Committee of Medical Journal Editors (2021). Defining the Role of Authors and Contributors. http://www.icmje.org/recommendations/browse/roles-and-responsibilities/defining-the-role-of-authors-and-contributors.html#two.

[8] Microbiology Society (2021). Open Access rates. https://www.microbiologyresearch.org/publishing-costs.

[9] Microbiology Society (2020). How to prepare an article for submission: Data. https://www.microbiologyresearch.org/prepare-an-article#12.

[10] Ayorinde Afolayan (2019). The Applied Bioinformatics and Public Health Conference with *Microbial Genomics*. Microbiology Society blog. https://microbiologysociety.org/blog/the-applied-bioinformatics-and-public-health-conference-with-microbial-genomics.html.

